# Coronavirus disease 2019 and future pandemics: Impacts on livestock health and production and possible mitigation measures

**DOI:** 10.14202/vetworld.2021.2434-2443

**Published:** 2021-09-20

**Authors:** Md. Hakimul Haque, Md. Aminul Islam, Md. Rezaul Karim, Mohammad Enamul Hoque Kayesh, Subir Sarker, K. H. M. Nazmul Hussain Nazir, M. Sawkat Anwer

**Affiliations:** 1 Department of Veterinary and Animal Sciences, Faculty of Agriculture, Rajshahi University, Rajshahi-6205, Bangladesh; 2 Department of Medicine, Faculty of Veterinary Medicine and Animal Science, Bangabandhu Sheikh Mujibur Rahman Agricultural University, Gazipur, 1706, Bangladesh; 3 Division of Animal Health Research, Bangladesh Livestock Research Institute, Savar, Dhaka-1341, Bangladesh; 4 Department of Microbiology and Public Health, Faculty of Animal Science and Veterinary Medicine, Patuakhali Science and Technology University, Barishal-8210, Bangladesh; 5 Department of Physiology, Anatomy and Microbiology, School of Life Sciences, La Trobe University, Melbourne, VIC 3086, Australia; 6 Department of Microbiology and Hygiene, Bangladesh Agricultural University, Mymensingh-2202, Bangladesh; 7 Department of Biomedical Sciences, Cummings School of Veterinary Medicine, Tufts University, North Grafton, Massachusetts 01536, USA.

**Keywords:** coronavirus disease 2019, impact, livestock, mitigation, poultry

## Abstract

The World Health Organization declared coronavirus disease 2019 (COVID-19) a pandemic on March 11, 2020. COVID-19, the current global health emergency, is wreaking havoc on human health systems and, to a lesser degree, on animals globally. The outbreak has continued since the first report of COVID-19 in China in December 2019, and the second and third waves of the outbreak have already begun in several countries. COVID-19 is expected to have adverse effects on crop production, food security, integrated pest control, tourism, the car industry, and other sectors of the global economy. COVID-19 induces a range of effects in livestock that is reflected economically since human health and livelihood are intertwined with animal health. We summarize the potentially harmful effects of COVID-19 on livestock and possible mitigation steps in response to this global outbreak. Mitigation of the negative effects of COVID-19 and future pandemics on livestock requires the implementation of current guidelines.

## Introduction

Globally, emerging and re-emerging pathogens pose a major threat to public health [1]. Coronavirus disease 2019 (COVID-19) was discovered in Wuhan, Hubei Province, China, at the end of 2019, and since then, COVID-19 has spread quickly across the world, causing millions of human infections and deaths [2]. The COVID-19 pandemic has become the greatest public health crisis of the 21^st^ century and creates serious health and socioeconomic challenges [3,4]. COVID-19 is caused by severe acute respiratory syndrome-coronavirus-2 (SARS-CoV-2) [5-7]. Coronavirus infections in humans and animals are not uncommon, and the virus has a broad host range. Such viral infections are reported in various avian and ­mammalian species [8,9]. Cattle and horses are susceptible to bovine coronavirus (a beta coronavirus) infection [10,11]. Chickens are the natural host for avian coronaviruses (CoVs) (a gamma coronavirus) [12,13]. Porcine epidemic diarrhea virus and transmissible gastroenteritis virus are known, and a new alpha coronavirus that causes swine acute diarrhea syndrome coronavirus in piglets is recently reported [14]. Other CoVs in pigs include porcine delta coronavirus and porcine hemagglutinating encephalomyelitis virus (a beta coronavirus) [15,16].

After the emergence of SARS-CoV in 2002 and Middle East respiratory syndrome coronavirus (MERS-CoV) in 2012, SARS-CoV-2 is the third zoonotic coronavirus that emerged in late 2019 to infect humans [17-19]. Rarely, CoVs can spread from animals to humans and then spread among humans, as happened in the case of SARS-CoV-2. It is believed that COVID-19 originated in bats [7], although an intermediate host has not been identified (WHO report 2021). Coronavirus sequences like SARS-CoV-2 were identified in Malayan pangolins (*Manis javanica*) [20]. In another study, Shi *et al*. [21] tested for SARS-CoV-2 infection in ferrets, livestock, and human companion animals. Ferrets and cats were susceptible to SARS-CoV-2, although dogs showed less susceptibility. Pigs, chickens, and ducks were not susceptible to SARS-CoV-2 infection. SARS-CoV-2 infection was also identified in dogs in Hong Kong [22]. Dogs and cats were susceptible to experimental SARS-CoV-2 infection in another study, but only cats shed viruses [23]. SARS-CoV-2 infection is also documented in mink [24], and two modes of transmission are confirmed for these animals [25]. Furthermore, a SARS-CoV-2 mink-associated variant strain was discovered in Denmark [26], prompting the Ministry of Environment and Food of Denmark to order the culling of all mink in the country (approximately 17 million animals) [27,28]. The Bronx Zoo in the United States confirmed a nondomestic SARS-CoV-2 natural infection in tigers and lions [29]. In South Africa, a zoo puma tested positive for SARS-CoV-2 after coming in contact with an infected handler [30]. Tree shrews [31], a successful laboratory animal, were found to be susceptible to SARS-CoV-2 infection in an experimental setting [32]. A recent experimental infection in cattle revealed moderate susceptibility [6]. Furthermore, an *in silico* study based on amino acid sequence suggested that primates are at high risk for SARS-CoV-2 infection, whereas other mammals fell into medium to very high-risk categories [33]. However, no evidence is available that animals play a significant role in transmitting SARS-CoV-2 infection to humans; the risk of a spread of COVID-19 from animals to humans is considered low [34]. Consequently, more research is needed for a better understanding of the animal–human interface to aid the formulation and implementation of preventive measures to combat future COVID-19 transmission [35].

There are four genera of CoVs, including Alphacoronaviruses, Betacoronaviruses, Gammacoronaviruses, and Deltacoronaviruses, that can infect humans and animals [36]. Since the 1970s, CoVs have been linked to diarrhea, respiratory disorders, nervous disorders, urinary tract infections, and systemic infections in various domestic animals, including cattle, sheep, deer, and horses [37]. SARS-CoV-2, a member of the Beta CoV genus [38,39], is thought to have jumped from an unknown animal reservoir to humans [40-42]. Bats have been suspected as natural hosts for SARS-CoV-2, yet the origin of the virus remains unknown [43].

The COVID-19 pandemic is wreaking havoc on every aspect of society, including healthcare and environmental protection [44-46]. COVID-19 has a serious effect in many developing countries, and the full health, social, and economic impact of the pandemic will not become clear until after the pandemic is over. The poverty rate in developing countries might double because of COVID-19 effects on various sectors, including livestock according to some experts [47]. COVID-19 outbreaks also negatively affect animal health and livestock production [48-51]. In turn, such effects can affect the supply of safe animal-derived foods for human consumption. This review aims to discuss the impacts of COVID-19 on animal health and development and explore countermeasures for mitigating the impacts on the basis of current literature and authors’ expertise. This review might help ensure animal health and, by extension, human health.

## Zoonotic Importance of COVID-19

To date, seven human CoVs (HCoVs) have been identified, including two alpha-CoVs (HCoV-229E and HCoV-NL63) and five beta-CoVs (HCoV-OC43, HCoV-HKU1, SARS-CoV, MERS-CoV, and SARS-CoV-2) [6,7,52]. All the seven HCoVs are zoonotic and originated from bats, rodents, or domestic animals. For SARS-CoV and MERS-CoV, bats are considered as the main hosts, whereas palm civet cats and dromedary camels are the intermediate hosts [53,54]. SARS-CoV-2, a relative of SARS-CoV and MERS-CoV, seems likely to have also passed from bats to humans through an intermediate host. However, the intermediate hosts and the mechanism of transmission are yet to be determined [55]. SARS-CoV-2 infects many mammals through angiotensin-converting enzyme-2 receptor, and the identification of intermediate hosts involved in human-to-animal or animal-to-animal transmission of SARS-CoV-2 is critical for reducing the risk of future COVID-19 outbreaks [33,56] (Table-1).

## Impact of COVID-19 Pandemic on Livestock Health and Production

The COVID-19 pandemic might affect animal health and welfare and wildlife conservation [49]. Recently, dogs, cats, tigers, lions, ferrets, and minks tested positive for SARS-CoV-2 infection after close contact with COVID-19 patients [50,57], suggesting possible human-to-animal transmission. However, SARS-CoV-2 infection is not transmissible to poultry, goats, rabbits, and guinea pigs [50,58]. A potential indirect negative effect of the pandemic on livestock health exists, including impacts to cattle, sheep, goats, and other livestock [50,59]. Hence, targeted monitoring of various aspects of animal production during the pandemic might provide important insight into the impact of COVID-19. The current pandemic situation has resulted in confinement and inactivity of farmers and caused financial shortfalls. Such difficulty may reduce resources for farming and the availability of veterinary care. A serious impact on overall animal health and production might follow [60,61]. Adverse effects of the pandemic on livestock health and production and potential mitigation strategies are discussed below.

## Immediate Consequences

The COVID-19 pandemic produced a significant downturn in industrial animal production and the associated supply chain. Many slaughterhouses and meat processing facilities worldwide emerged as major COVID-19 hotspots. For instance, at least 40% of infections occurred at a single Smithfield Foods slaughter plant in South Dakota during mid-April 2020 [62]. Consequently, much of the livestock industry shut down, and millions of animals were killed rather than slaughtered for human consumption [51,63]. Restricted movement of people hindered preventive measures against animal diseases [64]. This lack of attention to prevention may have increased the risk of zoonotic disease transmission from wildlife to livestock. Critical aspects of diseases that affect multiple host species are high transmissivity and bidirectionality with pathogens easily passing between wildlife and livestock [63]. Wildlife may share resources with livestock, including farm buildings, pastures, and water. The common use of such resources may encourage the spread of infectious diseases to livestock and farmers [64-66]. Deadly zoonotic infections, such as salmonellosis, brucellosis, ringworm, rabies, tuberculosis (TB), cryptosporidiosis, and Q fever, can spread among animals and then to humans [59]. These diseases are important concerns when humans interact with livestock, emphasizing the importance of everyday biosecurity and adherence to meticulous hygiene after each human–animal physical interaction [63]. Other immediate consequences, such as waning animal care or large numbers of farm animals, will increase the spread of infections [67]. A severe, long-term COVID-19 pandemic will compromise the capacity of local, state, and national veterinary services with devastating consequences.

## Long-term Consequences

The long-term effect of COVID-19 on livestock production will depend on impacts on farmer livelihoods and livestock services [68,69]. Furthermore, the effects of long-standing budget restrictions on ­economic emergencies can have an indirect and differential impact on disease eradication in different countries [49]. For example, the foot and mouth disease (FMD) outbreak in the United Kingdom was associated with the spread of *Mycobacterium bovis* infection to other livestock, in part because of halting the removal of contaminated cattle from the ecosystem due to a lack of funds [70]. Furthermore, a shortage of funds in the 1980s allowed TB prevalence in New Zealand cattle herds to increase dramatically [71]. Border areas in several countries saw a spread of tropical transboundary animal diseases, such as FMD, peste des petits ruminants virus, sheep and goat pox (SGP), and lumpy skin disease (LSD) [72-74]. Such tropical diseases spread to neighboring countries in various ways, including transport of diseased animals (e.g., PPR, SGP, and LSD) or naturally infected vectors (e.g., LSD) [49,72,75]. Since forest animals or vector-mediated access is likely, management and containment of diseases necessitate substantial investment of resources from veterinary services and farmers [49]. A global financial crisis directly or indirectly related to the COVID-19 pandemic severely limits the ability of farmers and veterinary facilities to invest needed funds [63], raising the possibility of an increased spread of transboundary diseases.

## Impact of COVID-19 on Livestock Production

The effects of coronavirus infections on livestock production remain largely unknown [50]. However, the following indirect effects of the pandemic might affect livestock production.

### Effects on veterinary care

Timely veterinary care from livestock experts is not always available because of lockdowns and restrictions on movement [49]. In addition, disruptions in various services essential for adequate production are expected (Figure-2). Livestock owners may face significant financial losses due to animal death, disease, and production losses (Figure-2) [64,75]. The pandemic has already delayed several international and national animal disease prevention and control programs, possibly jeopardizing global animal disease eradication programs [75].

**Figure-1:**
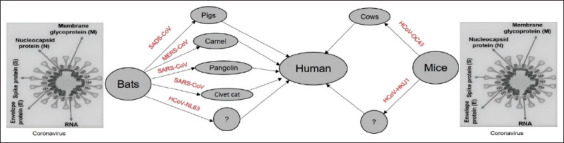
Transmission of coronavirus disease 2019 among animals and humans [modified from 56].

**Figure-2:**
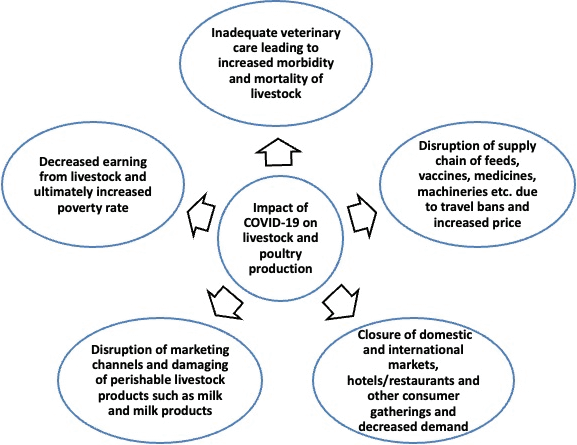
Impact of coronavirus disease 2019 on livestock health and production [64,75,80].

### Effects on animal feed

The efficient operation of the feed industry is likely to be affected by the pandemic. The inability to move freely during lockdown might result in labor shortages [64,76] and a decrease in the availability of materials for animal feeds [77]. Pastoralist transhumance is often hampered by movement restrictions, which makes it difficult for animal caregivers to adequately feed their livestock. The demand for animal feed is relatively stable, but the coronavirus crisis is expected to have a substantial effect on the supply of feed constituents (Figure-2), including soybeans, bakery ware, flour, corn, barley, and wheat [78]. Interference in the supply chain reduces feed resources even further [79]. In addition, the closing of restaurants globally has had a major impact on the animal feed industry. The SARS-CoV-2 epidemic prompted manufacturers to reconsider their policies and strategies because of the drastic change in ­consumer consumption habits [80]. The most affected industries are lamb and beef production. Meat intake has decreased dramatically around the world. In turn, the demand for grain and oilseed for animal feed decreased significantly [63]. The demand for soybean, corn, and wheat for animal feedstuff has decreased by approximately 6% in Vietnam, Thailand, Malaysia, and the Philippines [79]. The feed industry consumes 65% of the net annual production of soy, corn, and wheat, but 35% for the animal food industry [63].

### Effects on inputs and service access

Farmer access to breeding equipment and biological resources, such as sperm, may be limited because of restricted movement and disrupted supply chains, both national and international [59,77]. Pandemic-related disruption of livestock health inspections and extension programs is likely to result in renewed outbreaks of animal-related diseases. Vaccines and medications will be used more often to manage animal diseases in the future, resulting in higher vaccine and drug costs [80]. Import restrictions on livestock would have a significant impact on feedstuffs, biologics, vaccinations, and medicines [63,78], posing a risk to livestock health and productivity.

### Effects on marketing

The negative effects of COVID-19 on business supply chains can undermine marketing and food processing [51,81]. Many countries banned live animal markets, and many animals cannot be sold [77]. A recent study indicated that COVID-19 heavily impacted agro-livestock, farming, production, and marketing [76]. Trades are limited as a result of marketing network interruption and reduced consumer spending [60,78]. Farmers must maintain their goods for longer periods because of limited access to marketplaces and processing facilities. Higher processing and manufacturing costs and even real losses follow (Figure-2). Loss of income from small ruminant trade likely has a detrimental effect on women and their families by reducing their ability to buy household essentials [82]. Handling or vending is also disrupted by movement restrictions. Farmers can lose links to more influential purchasers as these intermediaries are disrupted. These issues have developed in previous pandemics. Many live animal markets are closed in several countries, and prices for small and large animals are reduced by more than half, forcing farmers to drastically reduce their production [63].

## Impact on Milk Production and Milk Price

The dairy industry also suffers during a pandemic. Millions of farmers depend on dairy farming for their livelihood, and the effects of COVID-19 on humans, dairy farmers, and the dairy industry as a whole are all important [83]. Some direct effects are failure to supply milk and milk products to customers and a reduction in the processing of milk and milk products. These reductions can lead to the closure of milk processing facilities, resulting in waste and disposal of large amounts of milk [49,63,84]. The risk of COVID-19 spread affects worldwide export and import of dairy products, including from China and other countries [63]. A viral outbreak increases the need for milk storage in central distributing countries, lowering global milk prices during the outbreak. Milk quality crises are already present in several countries because of the pandemic. Earlier this year, the dairy sector’s projected growth had slowed, which might have had the worst impact on the industry [78,83]. This trend is expected to continue for the rest of the year. Concurrently, milk prices, a major source of income for dairy farmers, are projected to decline. The COVID-19 outbreak presents no danger to dairy cattle; still, negative economic implications for the industry and farmers are likely to require a mitigation plan [85].

## Impact on the Poultry Sector

Chickens are resistant to SARS-CoV2 infection, but the COVID-19 pandemic is expected to affect the poultry industry because of disruption in the consumption and transport of poultry products [86]. Many hotels, restaurants, and fast-food outlets have closed as a precaution against the spread of COVID-19. Furthermore, social activities, such as weddings, have been postponed or canceled. These issues sharply reduce the demand for poultry, meat, and eggs (Figure-2) [80]. The demand for chickens and eggs plummeted as a result of government shutdowns. Hatcheries were forced to sell hatched eggs at throwaway prices or destroy chicks; prices of chicken, egg, and meat reached new lows [63]. According to industry reports, 14 million day-old chicks were hatched every week in Bangladesh before the coronavirus pandemic. This number has dropped as low as 7 million since the onset of COVID-19 [87]. The pandemic, according to this study, may have disastrous implications for other developing countries worldwide.

## Selling Poultry Products below Production Costs

Supply and demand imbalances in the global poultry industry harm the global poultry trade [86]. Egg and broiler chicken prices plunged, forcing farmers to offer poultry items below their production costs [80]. Farm eggs are being sold at farm level in some parts of Bangladesh for US $0.05 per egg, against a cost of production of at least US $0.07, whereas the average cost of a farm egg was US $0.08-0.09 before the shutdown began [87]. The price of broiler chickens had fallen to US $0.65 per kilogram at the farm level in various parts of Bangladesh when compared with the production costs of about US $1.41 per kilogram [87].

The poultry industry in many countries is thus bracing for massive losses caused by the COVID-19 pandemic and sales of poultry chicken and eggs have already fallen on the global market [80,88]. In the early days of the pandemic, Bangladesh saw a 12 year low in poultry prices, which harmed the livelihoods of millions of backyard poultry farmers and small traders [89]. Preventive steps are needed to minimize further losses because poultry farmers and entrepreneurs are currently unable to sell their goods [86]. The pandemic has also affected the global poultry trade. The top three exporters of poultry meat are the United States (4.1 Mt), Brazil (3.9 Mt), and the Netherlands (1.1 Mt), whereas the top importers are China, Hong Kong SAR (1.2 Mt), Japan (1.1 Mt), and Saudi Arabia (0.9 Mt) [90]. The ban on the movement of live poultry has limited the sale of poultry and eggs in Chinese markets. Chicken demand has been reduced by approximately 50% globally [91].

## Labor Shortage

A key problem in livestock production is the availability of labor, on the farm and in the supply chain, including hatcheries, catchers, feed mills, manufacturing, and packing centers [63]. In several parts of the world, logistical constraints and labor shortages threaten some agricultural market chains [59,82], restrict access to animal feeds, and reduce the capacity of slaughterhouses, processing plants, and other facilities. These issues place some food chains at risk [92]. Hussain *et al*. [78] reported a 24.7% labor shortage in broiler production in Pakistan as a result of transportation restrictions during the lockdown period. Furthermore, imports of feed ingredients, such as soybean meal and feed additives, as well as veterinary medicines, may be disrupted by the pandemic. The price of poultry and eggs will increase. The travel ban, particularly, has affected the distribution of poultry breeding stocks in several countries [80]. Travel restrictions might continue, and the International Poultry Council has warned that no breeding stock or hatching eggs will be left [93].

## Measures to be Taken to Protect Animal Health and Production

We must fully utilize the capabilities of veterinary epidemiologists and ecologists [80] to strengthen our understanding of SARS-CoV-2 and determine the most useful intervention/control steps. Careful attention should be paid to the following concerns.

### Marketing

**Figure-3:**
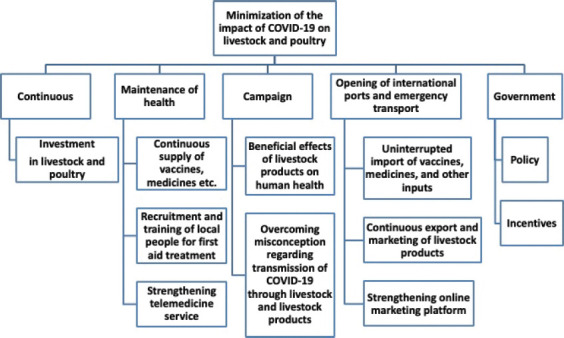
Mitigation approaches for COVID-19 impacts on livestock health and production [63,78,80].

Marketing is a critical aspect of the livestock industry, and proper marketing of livestock and associated products (milk, meat, hides and skin, and chicks) is necessary. Established and sustained, marketing channels are needed for consistent prices across the country [82]. Perishable livestock products, such as liquid milk, should be marketed in a timely fashion, and the government should regulate these products as emergency goods (Figure-3) [78]. Furthermore, commercial processors should restart or continue to process livestock products [82]. Additional efforts should also be made to leverage livestock products by alternative methods, such as powdered milk, freezing milk, meats, and other products, and by maintaining a strict cold chain. The smooth operation of the market chain requires drivers for transporting livestock goods, live livestock, poultry, and their feeds. This aspect of marketing should be granted special permission [59]. Drivers carrying livestock products must conform to the World Health Organization’s recommended precautions [63]. Farmers must adhere to the World Health Organization’s sanitary standards as well. Selling and purchasing live livestock and associated goods may need more emphasis on digital marketing/e-commerce.

### Campaign

Immunoglobulin (IgG) is a protein found in cow’s milk that helps defend against pathogens, including viruses, bacteria, and fungi, as well as allergens [94]. Vitamins A and D3 are found in milk, and both are essential for the enhancement of IgG function. Cow’s milk may be an important factor in modulating the immune function of the upper respiratory tract [94]. A public awareness campaign should be launched to encourage people and especially children to consume cow’s milk. A rumor that COVID-19 originated in and is spread by livestock products has circulated. A campaign should be launched to dispel this myth (Figure-3) [63].

### Proper import

The current pandemic has resulted in a sluggish import situation, which has led to the global scarcity of raw materials for animal feed [59]. Permission should be granted with appropriate safety protocols for the continuous import of animal feed, feed raw materials such as soy and maize, as well as medicines and vaccines from exporting countries [80]. Container and vessel supply and distribution have been ­continuous. With precautions, international ports should be opened as usual. Local processing of animal feeds such as green grass should be prioritized.

## Minimizing Effects on Livestock Feed

Human meat intake has declined as a result of COVID-19 with a corresponding increase in consumption of soybean, corn, and wheat. This change has adversely affected the availability of soybean, corn, and wheat for livestock [59]. Thus, steps should be taken to encourage meat consumption as before, increasing grain availability for animal feed. Technologies for long-term storage of green grasses in silage form should be developed for areas where high green grasses are grown. Additional steps should be taken, using advanced technology, for local development of key food ingredients such as corn and soya [80].

## Workers

The majority of workers in livestock and poultry industries travel from rural to urban areas in a country for work [82]. Workers in this sector began to return to their rural homes as a result of stringent lockdown policies, seriously affecting the management, processing, supply chains, and distribution of livestock and associated products [59,63]. Livestock workers must be hired locally, or other accommodations must be made for them (Figure-3).

## Role of Government

New protocols should be developed by governments to ensure the safety of farmers and those involved in the livestock industry and to support the continuous production and supply of livestock and products. The government should establish guidelines to sustain optimal livestock output and distribution to stakeholders and minimize impacts to farmers and workers (Figure-3) [80]. The Dutch government, for example, requested and completed required transactions electronically, for example, through the internet. Other countries should follow suit if their output or supply has been severely impaired. A telemedicine consultation service should be established or the current system improved for disease diagnosis and treatment of animals and for disease prevention [75,95]. Furthermore, each country must maintain critical activities, such as national and local veterinary regulatory and inspection services; food inspection and protection; emergency response; disease prevention measures, such as vaccination; and priority research activities [96]. Governments of all countries will need appropriate mitigation for the negative effects of COVID-19 on the livestock sector now, or the livestock sector will suffer along with other stakeholders involved in livestock. Impacts on the sector would last beyond the current COVID-19 pandemic [78].

## Financial Support

To alleviate the impact of the current global crisis on livestock production systems, low-interest loans, direct financial assistance [82], subsidies, and incentives should be made available to livestock enterprises [77] before the end of the COVID-19 crisis (Figure-3).

## Conclusion

COVID-19 should be viewed as a hazard to livestock health and production. The disease has a detrimental effect on buyer’s conduct and the ability of workers to care for livestock. COVID-19 can stymie livestock development by interfering with the transportation of livestock feeds, biologicals, and the marketing of livestock products, such as milk, ­poultry, eggs, hides, and skin. Appropriate intergovernmental and intragovernmental policies and successful rule enforcement are critical for minimizing the effects of COVID-19 on livestock output. Various livestock facilities, inputs, and goods transportation should be deemed emergency services. Implementing a telemedicine system for livestock service is crucial. Farmers impacted by the COVID-19 pandemic should be offered financial support as well as an introduction to e-commerce for livestock marketing. The current pandemic sends a strong message: Emerging and re-emerging pandemic crises not only impact human health directly but also put people at risk by affecting food security and economic stability and, eventually, by raising the likelihood of global hunger and famine. The world should be prepared to address human disasters by developing revolutionary new technologies that are smarter, more effective, and more widely applicable.

## Authors’ Contributions

MHH, MAI, MRK, and MEHK: Designed the study. MHH, MAI, MRK, and MEHK: Drafted the manuscript. MHH, MAI, MRK, MEHK, SS, KHMNHN, and MSA: Critically reviewed and updated the manuscript to its final version. The final version of the manuscript was approved by all authors.
